# Oxidative stress activates AMPK in cultured cells primarily by increasing cellular AMP and/or ADP

**DOI:** 10.1016/j.febslet.2014.07.025

**Published:** 2014-09-17

**Authors:** F. Romana Auciello, Fiona A. Ross, Naoko Ikematsu, D. Grahame Hardie

**Affiliations:** Division of Cell Signalling & Immunology, College of Life Sciences, University of Dundee, Dundee, DD1 5EH Scotland, UK

**Keywords:** AMPK, AMP-activated protein kinase, CaMKK, calmodulin-dependent kinase kinase, GO, glucose oxidase, LKB1, liver kinase B1, AMP, ADP, AMP-activated protein kinase, Hydrogen peroxide, Oxidative stress

## Abstract

AMPK is known to be activated by oxidative stress. Addition of glucose oxidase to cells generates H_2_O_2_ at a constant rate that is opposed by enzymic degradation, providing a good model for physiological oxidative stress. AMPK activation by glucose oxidase correlated with increases in cellular AMP:ATP and was greatly reduced in cells expressing an AMP-insensitive AMPK mutant, although a small degree of activation remained. The effects of increased AMP were partly due to inhibition of Thr172 dephosphorylation. These results suggest that changes in adenine nucleotides, rather than direct oxidative modification, are the major drivers of AMPK activation during oxidative stress.

## Highlights

• AMPK is activated by oxidative stress generated using glucose oxidase.
• Activation is largely, but not solely, mediated by increases in AMP and/or ADP.
• Increases in Thr172 phosphorylation are mediated by inhibition of dephosphorylation.

## 1 Introduction

The AMP-activated protein kinase (AMPK) is a sensor of cellular energy status, which occurs in all eukaryotes as heterotrimeric complexes comprising catalytic α subunits and regulatory β and γ subunits [1], [2], [3]. AMPK is activated by phosphorylation of Thr172 within the kinase domain by upstream kinases, with the principal upstream kinase being the tumor suppressor LKB1 [4], [5], [6], and/or by binding of allosteric activators at multiple sites [7]. Binding of AMP to the γ subunit, which is antagonized by ATP, activates AMPK by three complementary mechanisms: (i) allosteric activation; (ii) promotion of Thr172 phosphorylation by LKB1; (iii) inhibition of Thr172 dephosphorylation, which can also be triggered by binding of ADP [8], [9], [10], [11], [12]. Cellular stresses that inhibit ATP production or accelerate ATP consumption activate AMPK by causing increases in cellular AMP:ATP and ADP:ATP ratios [8], and AMPK then acts to restore energy homeostasis by switching on catabolic pathways generating ATP, while inhibiting ATP-consuming processes [1], [2], [3]. An alternative upstream activating pathway is triggered by increases in cellular Ca^2+^, causing Thr172 phosphorylation by the calmodulin-dependent protein kinase, CaMKKβ [13], [14], [15].

AMPK can also be activated by oxidative stress, usually triggered experimentally by adding reactive oxygen species such as H_2_O_2_ or NO to the cell medium [16]. Addition of H_2_O_2_ causes increases in cellular AMP:ATP, suggesting that AMPK activation is via the classical AMP-mediated pathway [17]. To confirm this, we constructed HEK-293 cell lines stably expressing either wild type AMPK (WT cells) or an AMP/ADP-insensitive mutant (RG cells). AMPK was activated by H_2_O_2_ in WT but not RG cells, while H_2_O_2_ inhibited oxygen uptake and increased ADP:ATP ratios in both; these results suggest that H_2_O_2_ activates AMPK by an AMP/ADP-dependent mechanism involving inhibition of the mitochondrial respiratory chain [18]. This was, however, challenged by a recent study in which H_2_O_2_ was generated in the medium by addition of glucose oxidase (GO); these authors presented evidence for an alternative mechanism involving oxidation of two conserved cysteine residues within the AMPK catalytic subunit [19]. We have therefore re-investigated the mechanism by which oxidative stress activates AMPK.

## 2 Materials and methods

### 2.1 Materials and proteins

GO from *Aspergillus niger*, catalase from bovine liver, A23187, H_2_O_2_ and anti-FLAG antibodies were from Sigma, and STO609 from Tocris Bioscience. A769662 was synthesized in-house [20]. Affinity-purified antibodies against AMPK-α subunits were described previously [21]. Phosphospecific anti-Thr172 antibodies were from Cell Signalling.

### 2.2 Cell culture

HEK-293 and HeLa cells were from ECACC/HPA (Porton Down, UK) and grown in DMEM containing 4.5 g/L glucose, 10% (v/v) fetal bovine serum (FBS), 100 IU/ml penicillin and 100 μg/ml streptomycin. HEK-293 cells expressing inducible human AMPK-γ2 subunits were generated as follows. DNA encoding full-length γ2 was amplified with primers designed to encode a 5′-BamHI site and a C-terminal FLAG tag, followed by an XhoI site. The resulting PCR product was cloned into the pcDNA5/FRT/TO plasmid (Invitrogen) to create the plasmid pcDND5/FRT/TO/γ2. The R531G mutation was created in this plasmid using the QuikChange Site-Directed Mutagenesis system (Stratagene). T-Rex HEK293 cells containing a single Flp recombinase target (FRT) site (Invitrogen) were transfected with Fugene6 (Promega) using the plasmids POG44 encoding Flp recombinase (Invitrogen) and pcDND5/FRT/TO/γ2 at a ratio of 9:1. After 48 h, cells were detached using trypsin and re-plated in medium containing hygromycin B (200 μg/ml) and blasticidin (15 μg/ml). Medium was replaced every 3 days until cell foci could be identified, and individual foci were then selected and expanded. Expression of AMPK-γ2 (WT or RG) was induced with tetracycline (1 μg/ml) for 48 h.

### 2.3 AMPK assays in cell lysates

Cell lysates (100 μg protein) were immunoprecipitated by incubation at 4 °C for 2 h on a roller mixer with 6 μl of anti-AMPKα1/α2 antibody coupled to protein G-Sepharose, and the immunoprecipitates assayed for AMPK using the AMARA peptide [22]. When AMPK activity was assayed in HEK-293 cells expressing recombinant FLAG-tagged γ2 subunit, immunoprecipitation was performed using 7 μl of EZview Red anti-FLAG M2 affinity gel from Sigma [18].

### 2.4 Western blotting and other analytical procedures

For analysis of ACC, SDS–PAGE was performed using Novex NuPAGE Tris–Acetate 3–8% gradient polyacrylamide gels in the Tris–Acetate buffer system. For other proteins, SDS–PAGE was performed using Novex NuPAGE Bis-Tris 4–12% gradient polyacrylamide gels in the MOPS buffer system (Invitrogen). Proteins were transferred to nitrocellulose membranes using the Xcell blot module (Biorad). Membranes were blocked in Li-Cor Odyssey blocking buffer for 1 h and scanned with the Li-Cor Odyssey IR imager using the appropriate secondary antibody coupled to IR680 or IR800 dye. H_2_O_2_ in cell media was estimated using the Cell Biolabs OxiSelect^™^ Hydrogen Peroxide Assay Kit. For the estimation of ADP:ATP ratio, cellular nucleotides were extracted in perchloric acid and analysed by capillary electrophoresis [18].

### 2.5 Statistical analysis

Statistical significance was assessed by 1-way ANOVA using GraphPad Prism 6, with Sidak’s multiple comparison test: ^∗^*p* < 0.05, ^∗∗^*p* < 0.01; ^∗∗∗^*p* < 0.001; ^∗∗∗∗^*p* < 0.0001.

## 3 Results

### 3.1 AMPK activation correlates with cell nucleotides when H_2_O_2_ is generated using glucose oxidase

**Fig. 1:**
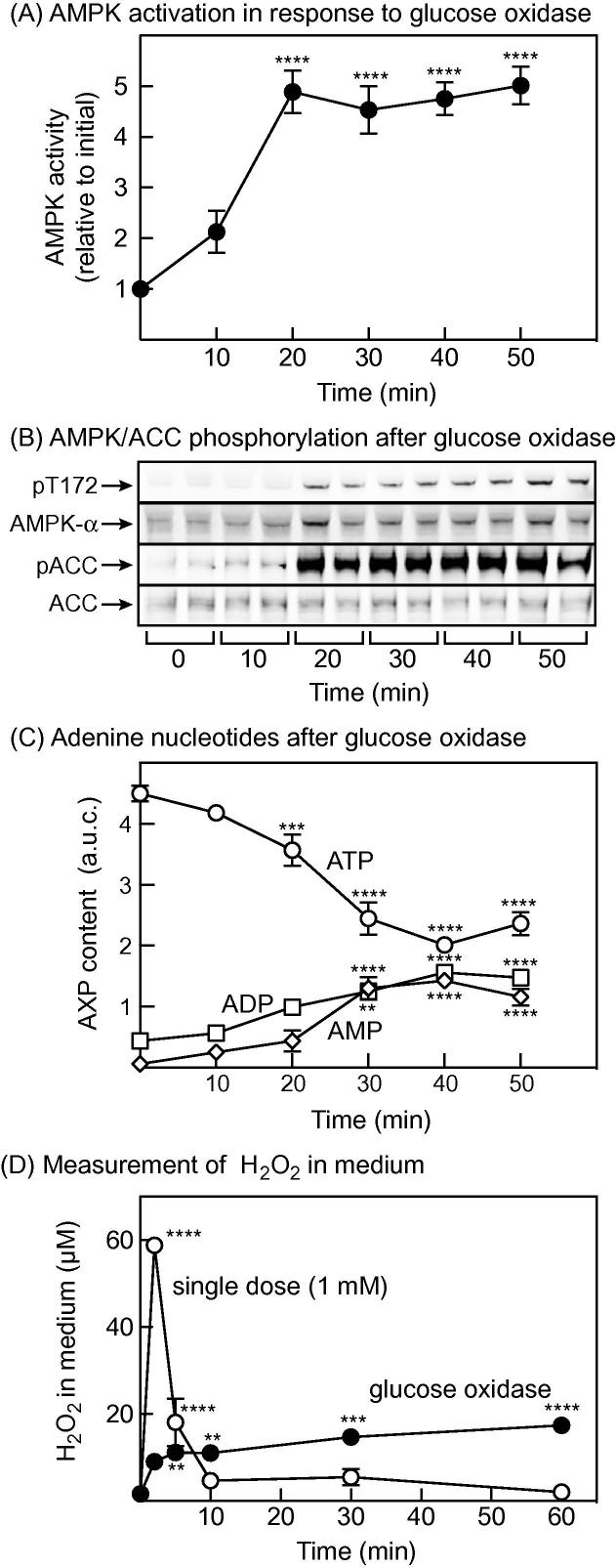
AMPK activation in response to GO treatment of HEK-293 cells, and measurement of H_2_O_2_ in the cell medium. (A) Activation of endogenous AMPK by GO (10 mU/ml) measured by immunoprecipitate kinase assay. Results are mean ± SEM (*n* = 12–18); mean values significantly different from the zero time value are indicated. (B) Phosphorylation of Thr172 on AMPK (pT172) and the AMPK site on acetyl-CoA carboxylase (pACC) at various times after addition of GO. Samples were from duplicate dishes from a single experiment. (C) Estimated contents of ATP, ADP and AMP at various times after addition of GO; values are area under curve (a.u.c.) of absorbance at 254 nm. Mean values significantly different from values at time zero are indicated (above the symbol for AMP and ATP, below for ADP; *n* = 3). (D) Analysis of H_2_O_2_ concentration in the cell medium at various times after addition of a single dose of H_2_O_2_ (calculated to give a final concentration of 1 mM) or GO (10 mU/ml). Mean values significantly different from values at time zero are indicated (*n* = 4). Statistical significance was assessed by 1-way ANOVA using Sidak’s multiple comparison test: ^∗^*p* < 0.05, ^∗∗^*p* < 0.01, ^∗∗∗^*p* < 0.001, ^∗∗∗∗^*p* < 0.0001.

Pilot experiments revealed that addition of GO at 5 mU/ml or less to HEK-293 cells did not cause significant changes in ADP:ATP ratio, AMPK activity or phosphorylation of the downstream target acetyl-CoA carboxylase (ACC), presumably because any H_2_O_2_ produced was immediately broken down by cellular enzymes. However, at 10 mU/ml we observed AMPK activation and Thr172 phosphorylation, and a marked phosphorylation of ACC, which were maximal by 20 min and then stable for up to 50 min (Fig. 1A and B). When we estimated the cellular contents of adenine nucleotides, there were decreases in ATP and increases in AMP and ADP that became significant by 20–30 min, which then remained relatively constant up to 50 min (Fig. 1C). As expected [8], the increases in AMP (≈20-fold) were larger than the increases in ADP (3- to 4-fold) or the decreases in ATP (≈2-fold). Thus, AMPK activation and ACC phosphorylation showed a temporal correlation with the increases in AMP and ADP, and the decrease in ATP, during GO treatment.

When we measured H_2_O_2_ concentration in the medium following addition of 10 mU/ml GO, it increased to 10 μM within 5 min, and then more gradually to around 20 μM by 60 min. By contrast, when a single dose of H_2_O_2_ was added (to a calculated final concentration of 1 mM), the actual H_2_O_2_ measured in the medium was only 60 μM at the first time point (2 min), had dropped to <5 μM by 10 min, and was undetectable by 60 min (Fig. 1D). Thus, a single dose of H_2_O_2_ is metabolized very rapidly by HEK-293 cells, whereas when GO is added a quasi-steady state is reached within 5 min where the rate of H_2_O_2_ production is balanced by its breakdown.

### 3.2 Effect of GO in cells expressing an AMP-insensitive AMPK mutant

**Fig. 2:**
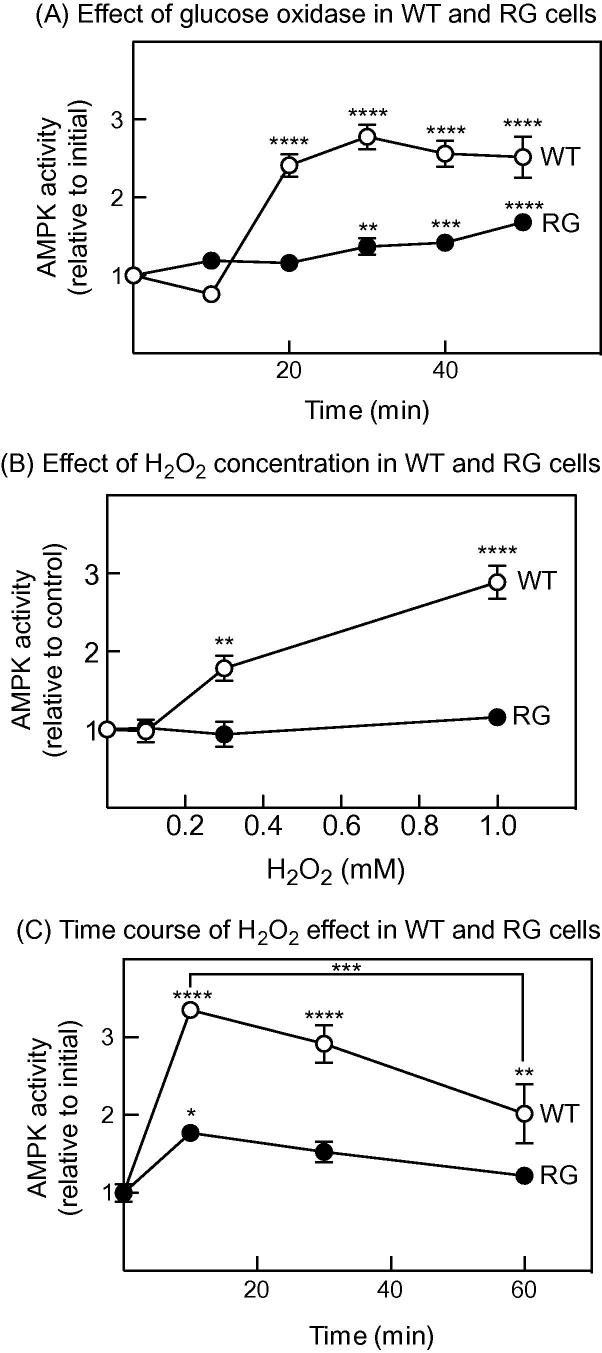
Activation of WT AMPK and the AMP/ADP-insensitive RG mutant by GO and H_2_O_2_. (A) Activation of AMPK in response to GO (10 mU/ml) measured in anti-FLAG immunoprecipitates in WT and RG cells. Results are mean ± SEM (*n* = 20–24). (B) As (A), but adding a single dose of H_2_O_2_ at the calculated final concentration shown, and then preparing cell lysates 60 min later. Results are mean ± SEM (*n* = 4). (C) As (B), but varying the time of incubation with a calculated final concentration of 1 mM H_2_O_2_. Results are mean ± SEM (*n* = 4). Statistical significance was assessed by 1-way ANOVA using Sidak’s multiple comparison test: ^∗^*p* < 0.05, ^∗∗^*p* < 0.01, ^∗∗∗^*p* < 0.001, ^∗∗∗∗^*p* < 0.0001.

To test whether AMPK activation by GO was mediated by increases in AMP or ADP, we examined its effects in HEK-293 cells expressing FLAG-tagged AMPK-γ2, either wild type (WT cells) or the AMP/ADP-insensitive R531G mutant (RG cells). These were similar to those used previously [18] except that AMPK-γ2 was expressed from a tetracycline-inducible promoter. When we treated WT cells with GO, there was a large activation (2.5- to 3-fold) of AMPK in anti-FLAG immunoprecipitates between 10 and 20 min that was sustained up to 50 min, similar to the results with endogenous AMPK in Fig. 1. By contrast, there was no activation in RG cells by 20 min, although there was a significant activation at later time points (Fig. 2A).

We also re-examined the effect of adding a single dose of H_2_O_2_ to the WT and RG cells. Fig. 2B shows the dependence of AMPK activity on H_2_O_2_ concentration, measured 60 min after addition, while Fig. 2C shows the time course with 1 mM H_2_O_2_. Note that the H_2_O_2_ concentrations in Fig. 2B are calculated, although Fig. 1D suggests that the actual concentrations decline very rapidly following addition to cells. Activation of WT AMPK was observed 60 min after addition of H_2_O_2_ to 300 μM or 1 mM, but not 100 μM (Fig. 2B). As previously reported [18], no activation was observed 60 min after addition with the RG mutant at any H_2_O_2_ concentration. However, some activation by 1 mM H_2_O_2_ was seen with the RG mutant after 10 min, which then declined to baseline by 60 min. Maximal activation of AMPK in WT cells was observed at 10 min after which it declined, although >2-fold activation was still observed at 60 min (Fig. 2C).

### 3.3 Effect of catalase and the CaMKK inhibitor, STO609

**Fig. 3:**
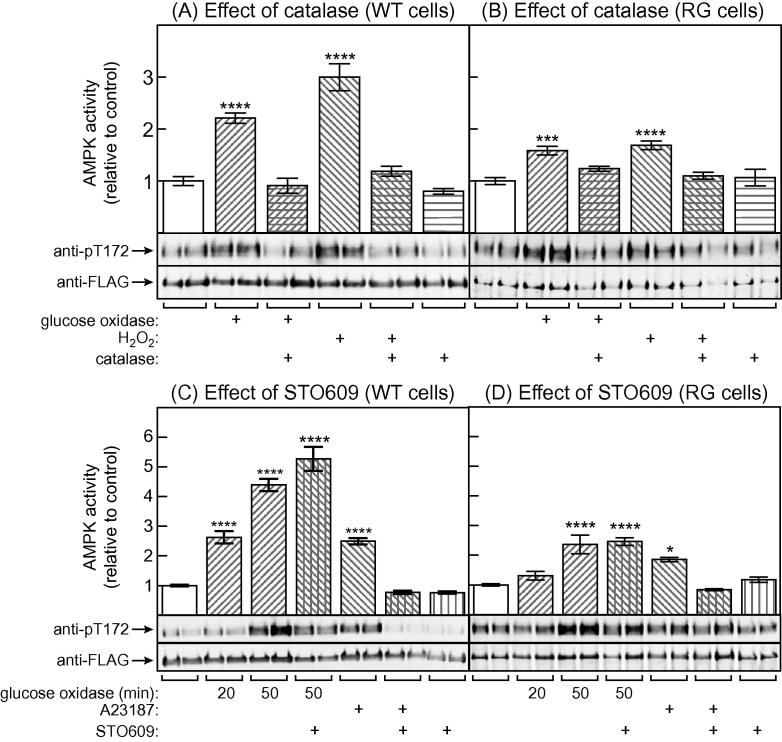
Effect of catalase and STO609 on AMPK activation of WT AMPK and the RG mutant by GO and H_2_O_2_. (A) Catalase (5 U/ml) was added to HEK-293 cells expressing FLAG-tagged WT γ2 (WT cells); 2 h later GO (10 mU/ml) or H_2_O_2_ (calculated final concentration 1 mM) was added and 50 or 10 min later (respectively) cell lysates and anti-FLAG immunoprecipitates were prepared for AMPK assay. Results in the upper panel are mean ± SEM (*n* = 4). The lower panels show blots of duplicate dishes of cells analyzed for Thr172 phosphorylation and FLAG-γ2 expression. (B) As (A), but using RG cells. Results in the upper panel are mean ± SEM (*n* = 4). (C) As (A), but adding the CaMKK inhibitor (STO609 (25 μM)) instead of catalase, 1 h before addition of GO; lysates were prepared for AMPK assay 20 or 50 min later, as indicated. Where indicated, A23187 (10 μM) was added 30 min prior to cell lysates being prepared. Results in the upper panel are mean ± SEM (*n* = 12–18). (D) As (C), but using RG rather than WT cells. Results in the upper panel are mean ± SEM (*n* = 6–12). Statistical significance was assessed by 1-way ANOVA using Sidak’s multiple comparison test: ^∗^*p* < 0.05, ^∗∗^*p* < 0.01, ^∗∗∗^*p* < 0.001, ^∗∗∗∗^*p* < 0.0001.

To confirm that the effect of GO was mediated by generation of H_2_O_2_, we pre-treated WT and RG cells with catalase prior to addition of GO or H_2_O_2,_ and then measured AMPK activity and Thr172 phosphorylation 50 min later (Fig. 3A and B). The results showed that catalase alone had no effect, but that it abolished the large increases in AMPK activity and Thr172 phosphorylation observed in response to GO or H_2_O_2_ in the WT cells, as well as the much smaller increases observed in the RG cells. We also pretreated WT and RG cells with the CaMKK inhibitor STO609 [23], and then incubated with GO or the Ca^2+^ ionophore A23187 for 50 min. Although STO609 abolished the effect of A23187, it did not affect the response to GO in either WT or RG cells, showing that increased AMPK activity and Thr172 phosphorylation was not dependent on CaMKKβ under these conditions.

### 3.4 H_2_O_2_ treatment inhibits Thr172 dephosphorylation in intact cells

**Fig. 4:**
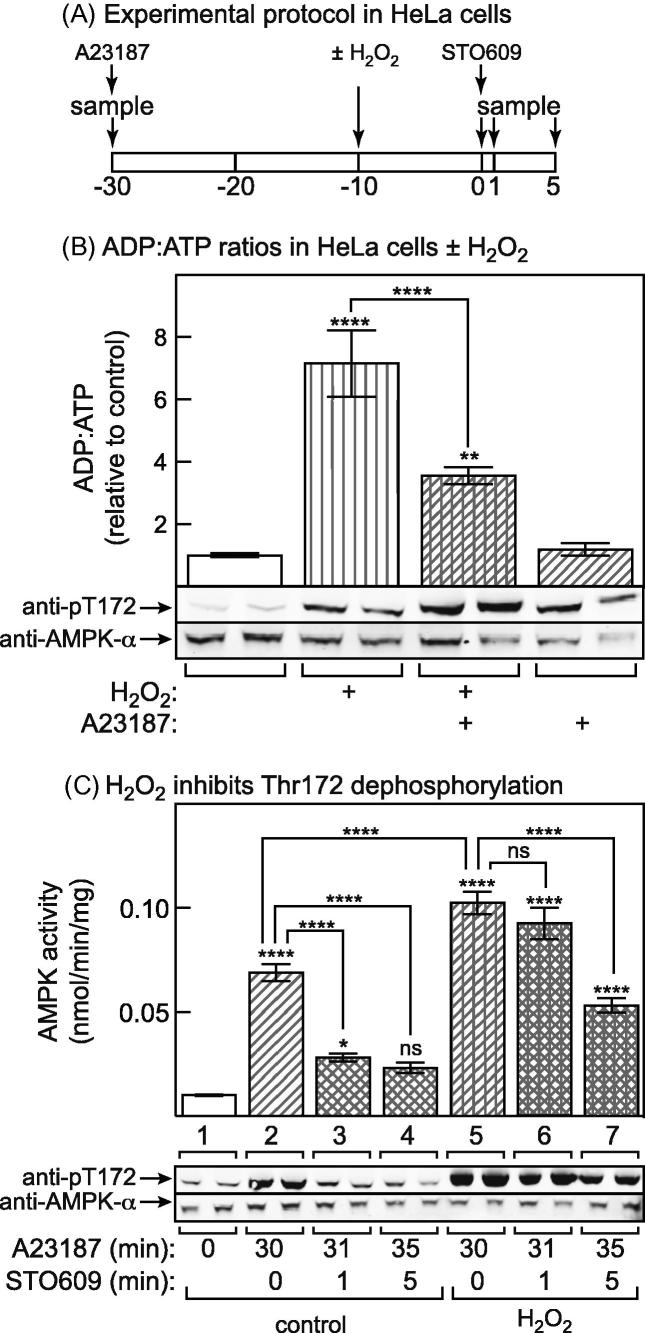
H_2_O_2_ promotes Thr172 phosphorylation by inhibiting dephosphorylation. (A) Protocol for assays to monitor Thr172 dephosphorylation in intact cells. (B) HeLa cells were incubated with or without 10 μM A23187 for 30 min, with or without H_2_O_2_ (calculated concentration 1 mM) added for the last 10 min. Nucleotides were then extracted for analysis of ADP:ATP ratios. (C) Activity (upper panel) and Thr172 phosphorylation (lower panel, duplicate dishes) of endogenous AMPK in HeLa cells during the protocol shown in (A). Lane 1 is basal activity and Thr172 phosphorylation at the start of the experiment. Lane 2 (control) and 5 (H_2_O_2_-treated) show activity and Thr172 phosphorylation 30 min after addition of A23187, lanes 3 or 4 (control) and 6 or 7 (H_2_O_2_-treated) show activity and Thr172 phosphorylation 1 or 5 min, respectively, after addition of STO609. The upper panel shows AMPK activity as mean ± SEM (*n* = 18). Mean values significantly different from the basal value (lane 1) are indicated immediately above each bars; significance of other differences are indicated above brackets linking bars. The lower panel shows blots from duplicate dishes. Statistical significance was assessed by 1-way ANOVA using Sidak’s multiple comparison test: ^∗^*p* < 0.05, ^∗∗^*p* < 0.01, ^∗∗∗^*p* < 0.001, ^∗∗∗∗^*p* < 0.0001.

We suspected that the increased Thr172 phosphorylation observed in response to H_2_O_2_ was mediated by binding of AMP to the AMPK-γ subunit, leading to inhibition of Thr172 dephosphorylation. To address this, we switched to HeLa cells. Due to the complete absence of LKB1 in these cells, basal phosphorylation of Thr172 is extremely low, but can be increased by addition of the Ca^2+^ ionophore A23187, which activates CaMKKβ [13]. Pilot experiments revealed that Thr172 phosphorylation had reached a new steady state 30 min after A23187 addition. At that point we added enough STO609 to completely inhibit CaMKKβ, providing an opportunity to measure the rate of Thr172 dephosphorylation in intact cells. This experimental protocol is summarized in Fig. 4A.

We first examined the effect of H_2_O_2_ and A23187 on adenine nucleotide ratios. Fig. 4B shows that treatment with 1 mM H_2_O_2_ for 10 min increased the cellular ADP:ATP ratio by 7-fold. Interestingly, this was reduced to 3- to 4-fold if AMPK had been activated by adding A23187 prior to H_2_O_2_, although A23187 had no effect on its own.

In HeLa cells not treated with H_2_O_2_, treatment with A23187 for 30 min caused a large AMPK activation and Thr72 phosphorylation, as expected (Fig. 4C, lanes 1 and 2). Interestingly, in cells to which H_2_O_2_ had been added 20 min after A23187, a higher steady state level of AMPK activation and Thr172 phosphorylation was observed (compare lanes 2 and 5). When STO609 was added to block CaMKKβ, there was an extremely rapid AMPK inactivation, concomitant with Thr172 dephosphorylation, in cells not pre-treated with H_2_O_2_. However, both effects were markedly reduced in the cells pre-treated with H_2_O_2_, which was particularly evident in the samples taken 1 min after addition of STO609.

## 4 Discussion

These results resolve some of the discrepancies between our previous study [18] and that of Zmijewski et al. [19]. We reported that WT AMPK was markedly activated by a singe dose of H_2_O_2_, whereas there was no activation of the RG mutant, and therefore concluded that the effect of H_2_O_2_ was mediated entirely through changes in cellular adenine nucleotides [18]. However, we had used a standard incubation time of 60 min for all AMPK activators examined in our study, and had not realized how rapidly a single dose of H_2_O_2_ is metabolized by cells (see Fig. 1D). Our present results (Fig. 2C) show that H_2_O_2_ did cause a small but significant activation of the RG mutant after 10 min, but that this had reverted to baseline by 60 min, which is why we previously missed it. The much larger activation observed with WT AMPK also declined between 10 and 60 min but was still evident at 60 min, as reported previously [18]. The reversal of AMPK activation between 10 and 60 min is presumably because the added H_2_O_2_ has been almost completely metabolized within 10 min (Fig. 1D), and because cellular anti-oxidant systems are able to partially reverse any oxidative damage within this timeframe.

Zmijewski et al. [19] used the alternative approach of adding GO, which generates H_2_O_2_ as a by-product of metabolism of medium glucose. The advantage of this is that, after a brief lag, the rate of production of H_2_O_2_ by GO is balanced by its destruction by cellular enzymes, so that a quasi-steady state is reached where the concentration of H_2_O_2_ in the medium remains approximately constant (Fig. 1D). This probably represents a better model of physiological oxidative stress than addition of a single high dose of H_2_O_2_. After a lag of 10–20 min, GO activated wild type AMPK by up to 5-fold, accompanied by increased phosphorylation of its downstream target ACC, and correlating with increases in AMP and ADP and decreases in ATP (Fig. 1). AMPK activation in response to GO was much lower in RG cells, although there was a small but significant activation after 30 min (Fig. 2A). The activation of the RG mutant we observed after 10 min treatment with a single dose of H_2_O_2_ (Fig. 2C), or after 30–60 min with GO (Fig. 2A) might have resulted from the cysteine oxidation mechanism proposed by Zmijewski et al. [19]. However, this effect is small compared with the AMP-dependent effect (compare the activation of the WT and RG mutants in Fig. 2). One remaining discrepancy between our results and Zmijewski et al. [19] is that they reported that incubation of HEK-293 cells with GO for 10–40 min (which caused AMPK activation and ACC phosphorylation) was not associated with changes in cellular ADP:ATP ratios. The reasons for this discrepancy remain unclear, in part because Zmijewkski et al. cited papers describing two different methods for extracting adenine nucleotides, and it is not clear which method they used. An additional difficulty is that they did not report absolute ADP:ATP ratios, but merely expressed the ratios relative to the control. It is therefore difficult to make direct comparisons between their nucleotide measurements and ours.

Our results confirm that the effects of adding H_2_O_2_ or GO on AMPK activity and Thr172 phosphorylation are mediated by H_2_O_2_, because both were abolished by pre-treatment of cells with catalase (Fig. 3A and B). In HEK293 cells, the upstream kinase producing the increased Thr172 phosphorylation was LKB1 rather than CaMKKβ, because the effect of GO was not sensitive to STO609 (Fig. 3C and D). However, the results in HeLa cells in Fig. 4 reveal that H_2_O_2_ can still promote Thr172 phosphorylation and AMPK activation when CaMKKβ is the upstream kinase, showing that the effect is independent of the upstream kinase being utilized. We have previously shown by measuring cellular oxygen uptake that H_2_O_2_ inhibits the function of the mitochondrial respiratory chain [18], and we propose that this causes increases in AMP or ADP (Fig. 4B) that bind to the AMPK-γ subunit, triggering conformational changes that inhibit Thr172 dephosphorylation. Since HEK-293 and HeLa cells display rapid glycolysis, additional possibilities are that DNA damage induced by oxidative stress depletes cellular NAD^+^ via activation of poly ADP-ribose polymerase (PARP) [24], or that oxidative stress promotes ADP-ribosylation of glyceraldehyde-3-phosphate dehydrogenase [25], both of which would inhibit glycolysis.

Because of the lack of LKB1 and the low basal activity of CaMKKβ, basal Thr172 phosphorylation and AMPK activity in HeLa cells is very low. However, addition of the Ca^2+^ ionophore A23187 caused AMPK activation and Thr172 phosphorylation, which reached a steady state within 30 min, when Thr172 phosphorylation by CaMKKβ was balanced by dephosphorylation. If the CaMKK inhibitor STO609 was then added, we could directly observe dephosphorylation within the intact cell in the absence of simultaneous phosphorylation. Interestingly, without H_2_O_2_ treatment this dephosphorylation appeared to be almost complete 1 min after STO609 addition, showing that the phosphate on Thr172 was turning over extremely rapidly. However, when H_2_O_2_ was added 10 min prior to STO609, an initial increase in steady state AMPK activation and Thr172 phosphorylation was observed, and then the rate of dephosphorylation on STO609 addition was drastically reduced. We suggest that this was primarily due to inhibition of dephosphorylation due to binding of AMP or ADP to the AMPK-γ subunit, which would explain both the increase in steady state AMPK activation/Thr172 phosphorylation prior to STO609 addition, and the reduced rate of inactivation/dephosphorylation following its addition. We cannot, however, rule out the additional possibility that protein phosphatase(s) acting on Thr172 are directly inhibited by oxidative stress.

Interestingly, while H_2_O_2_ caused increases in cellular ADP:ATP ratio in HeLa cells, they were significantly smaller in cells pre-treated with A23187 for 20 min (Fig. 4B). This could be explained if AMPK activation by A23187 caused metabolic changes that better prepared the cells for the energy stress caused by subsequent addition of H_2_O_2_. AMPK activation has been previously shown to induce expression of target genes for FOXO3, including several proteins involved in resistance to oxidative stress [26]. However, the effect we observed occurred after only 20 min, and changes not requiring transcription and translation, such as acute activation of glucose uptake and glycolysis [2] may perhaps be more likely to explain it.

While this study was in progress, a potential mechanism was proposed by which AMPK is *inactivated* (rather than activated) by oxidative stress in cardiac myocytes [27]. By over-expressing a substrate-trapping mutant of thioredoxin-1 (Trx1) in mouse heart, AMPK-α subunits were identified as major intracellular targets of Trx1. AMPK activation during ischemia was abolished in hearts of transgenic mice expressing Trx1 with mutations in its critical disulfide, and two conserved cysteine residues in the AMPK-α subunit (Cys130 and Cys174) were identified whose reduction by Trx1 prevented AMPK inactivation during glucose deprivation in cardiac myocytes, or cardiac ischaemia in vivo. These cysteines are within the kinase domain (Cys174 is almost adjacent to Thr172) and are distinct from those that Zmijewski et al. [19] suggested to be involved in AMPK *activation* during oxidative stress. Given the crucial requirement for AMPK in the response to cardiac ischaemia, it was suggested that Trx1 was an essential cofactor that protects against oxidative inactivation of AMPK during ischemia [27]. Interestingly, they did not find any evidence for oxidative modification of AMPK when HEK-293 cells were incubated with 300 μM H_2_O_2_ for 30 min, although Thr172 phosphorylation increased, presumably due to increased AMP inhibiting dephosphorylation as shown here. The authors speculated that, compared with primary cells, immortalized cell lines may have elevated levels of anti-oxidant defense systems that prevent inactivation of AMPK during oxidative stress, thus allowing mechanisms causing activation to become apparent [27].

## References

[1] Hardie D.G., Ross F.A., Hawley S.A. (2012). AMP-activated protein kinase: a target for drugs both ancient and modern. Chem. Biol..

[2] Hardie D.G., Ross F.A., Hawley S.A. (2012). AMPK: a nutrient and energy sensor that maintains energy homeostasis. Nat. Rev. Mol. Cell Biol..

[3] Hardie D.G. (2013). AMPK: a target for drugs and natural products with effects on both diabetes and cancer. Diabetes.

[4] Hawley S.A., Boudeau J., Reid J.L., Mustard K.J., Udd L., Makela T.P., Alessi D.R., Hardie D.G. (2003). Complexes between the LKB1 tumor suppressor, STRADa/b and MO25a/b are upstream kinases in the AMP-activated protein kinase cascade. J. Biol..

[5] Woods A., Johnstone S.R., Dickerson K., Leiper F.C., Fryer L.G., Neumann D., Schlattner U., Wallimann T., Carlson M., Carling D. (2003). LKB1 is the upstream kinase in the AMP-activated protein kinase cascade. Curr. Biol..

[6] Shaw R.J., Kosmatka M., Bardeesy N., Hurley R.L., Witters L.A., DePinho R.A., Cantley L.C. (2004). The tumor suppressor LKB1 kinase directly activates AMP-activated kinase and regulates apoptosis in response to energy stress. Proc. Natl. Acad. Sci. USA.

[7] Scott J.W., Ling N., Issa S.M., Dite T.A., O’Brien M.T., Chen Z.P., Galic S., Langendorf C.G., Steinberg G.R., Kemp B.E., Oakhill J.S. (2014). Small molecule drug A-769662 and AMP synergistically activate naive AMPK independent of upstream kinase signaling. Chem. Biol..

[8] Gowans G.J., Hawley S.A., Ross F.A., Hardie D.G. (2013). AMP is a true physiological regulator of AMP-activated protein kinase by both allosteric activation and enhancing net phosphorylation. Cell Metab..

[9] Hawley S.A., Selbert M.A., Goldstein E.G., Edelman A.M., Carling D., Hardie D.G. (1995). 5’-AMP activates the AMP-activated protein kinase cascade, and Ca^2+^/calmodulin the calmodulin-dependent protein kinase I cascade, via three independent mechanisms. J. Biol. Chem..

[10] Davies S.P., Helps N.R., Cohen P.T.W., Hardie D.G. (1995). 5’-AMP inhibits dephosphorylation, as well as promoting phosphorylation, of the AMP-activated protein kinase. Studies using bacterially expressed human protein phosphatase-2Ca and native bovine protein phosphatase-2A_C_. FEBS Lett..

[11] Zhang Y.L., Guo H., Zhang C.S., Lin S.Y., Yin Z., Peng Y., Luo H., Shi Y., Lian G., Zhang C., Li M., Ye Z., Ye J., Han J., Li P., Wu J.W., Lin S.C. (2013). AMP as a low-energy charge signal autonomously initiates assembly of AXIN–AMPK–LKB1 complex for AMPK activation. Cell Metab..

[12] Xiao B., Sanders M.J., Underwood E., Heath R., Mayer F.V., Carmena D., Jing C., Walker P.A., Eccleston J.F., Haire L.F., Saiu P., Howell S.A., Aasland R., Martin S.R., Carling D., Gamblin S.J. (2011). Structure of mammalian AMPK and its regulation by ADP. Nature.

[13] Hawley S.A., Pan D.A., Mustard K.J., Ross L., Bain J., Edelman A.M., Frenguelli B.G., Hardie D.G. (2005). Calmodulin-dependent protein kinase kinase-beta is an alternative upstream kinase for AMP-activated protein kinase. Cell Metab..

[14] Woods A., Dickerson K., Heath R., Hong S.P., Momcilovic M., Johnstone S.R., Carlson M., Carling D. (2005). Ca^2+^/calmodulin-dependent protein kinase kinase-beta acts upstream of AMP-activated protein kinase in mammalian cells. Cell Metab..

[15] Hurley R.L., Anderson K.A., Franzone J.M., Kemp B.E., Means A.R., Witters L.A. (2005). The Ca^2+^/calmodulin-dependent protein kinase kinases are AMP-activated protein kinase kinases. J. Biol. Chem..

[16] Cardaci S., Filomeni G., Ciriolo M.R. (2012). Redox implications of AMPK-mediated signal transduction beyond energetic clues. J. Cell Sci..

[17] Choi S.L., Kim S.J., Lee K.T., Kim J., Mu J., Birnbaum M.J., Soo Kim S., Ha J. (2001). The regulation of AMP-activated protein kinase by H_2_O_2_. Biochem. Biophys. Res. Commun..

[18] Hawley S.A., Ross F.A., Chevtzoff C., Green K.A., Evans A., Fogarty S., Towler M.C., Brown L.J., Ogunbayo O.A., Evans A.M., Hardie D.G. (2010). Use of cells expressing gamma subunit variants to identify diverse mechanisms of AMPK activation. Cell Metab..

[19] Zmijewski J.W., Banerjee S., Bae H., Friggeri A., Lazarowski E.R., Abraham E. (2010). Exposure to hydrogen peroxide induces oxidation and activation of AMP-activated protein kinase. J. Biol. Chem..

[20] Iyengar R.R., Zhao G., Judd A.S., Kifle L., Cao N.N., Chiou W.J., Cool B.L., Camp H.S., Frevert E.U., Turner T.M., Liu J.R., Huang Y., Marsh K.C., Mika A.K., Perham M.A., Zinker B.A., Sham H.L., Kym P.R. (2005). Discovery and SAR studies of thienopyridones: a class of small molecule AMPK activators. Abst. Papers Am. Chem. Soc..

[21] Woods A., Salt I., Scott J., Hardie D.G., Carling D. (1996). The a1 and a2 isoforms of the AMP-activated protein kinase have similar activities in rat liver but exhibit differences in substrate specificity *in vitro*. FEBS Lett..

[22] Towler M.C., Fogarty S., Hawley S.A., Pan D.A., Martin D.M., Morrice N.A., McCarthy A., Galardo M.N., Meroni S.B., Cigorraga S.B., Ashworth A., Sakamoto K., Hardie D.G. (2008). A novel short splice variant of the tumour suppressor LKB1 is required for spermiogenesis. Biochem. J..

[23] Tokumitsu H., Inuzuka H., Ishikawa Y., Ikeda M., Saji I., Kobayashi R. (2002). STO-609, a specific inhibitor of the Ca^2+^/calmodulin-dependent protein kinase kinase. J. Biol. Chem..

[24] Kim M.Y., Zhang T., Kraus W.L. (2005). Poly(ADP-ribosyl)ation by PARP-1: ‘PAR-laying’ NAD+ into a nuclear signal. Genes Dev..

[25] Dimmeler S., Lottspeich F., Brune B. (1992). Nitric oxide causes ADP-ribosylation and inhibition of glyceraldehyde-3-phosphate dehydrogenase. J. Biol. Chem..

[26] Greer E.L., Oskoui P.R., Banko M.R., Maniar J.M., Gygi M.P., Gygi S.P., Brunet A. (2007). The energy sensor AMP-activated protein kinase directly regulates the mammalian FOXO3 transcription factor. J. Biol. Chem..

[27] Shao D., Oka S., Liu T., Zhai P., Ago T., Sciarretta S., Li H., Sadoshima J. (2014). A redox-dependent mechanism for regulation of AMPK activation by thioredoxin1 during energy starvation. Cell Metab..

